# The Middle East Respiratory Syndrome Coronavirus (MERS-CoV) Outbreak at King Abdul-Aziz Medical City-Riyadh from Emergency Medical Services Perspective

**DOI:** 10.1017/S1049023X20000709

**Published:** 2020-05-20

**Authors:** Abdullah Alabdali, Kharsan Almakhalas, Faisal Alhusain, Saad Albaiz, Khalid Almutairi, Nawfal Aljerian

**Affiliations:** 1. Emergency Medical Services Department, College of Applied Medical Sciences, King Saud bin Abdulaziz University for Health Sciences, Riyadh, Saudi Arabia; 2. King Abdullah International Medical Research Center, Riyadh, Saudi Arabia; 3. Royal Commission Hospital, Jubail, Saudi Arabia; 4. Emergency Medicine Department, Ministry of National Guard Health Affairs, Riyadh, Saudi Arabia; 5. National Health Command Center, Ministry of Health, Riyadh, Saudi Arabia

**Keywords:** disaster, Emergency Medical Services, Middle East Respiratory Syndrome Coronavirus, paramedics, personal protective equipment

## Abstract

Middle East Respiratory Syndrome Coronavirus (MERS-CoV) is a form of an infectious respiratory disease, discovered in November 2012 in Saudi Arabia. According to the World Health Organization (WHO; Geneva, Switzerland) reports, a total of 2,519 laboratory-confirmed cases and 866 MERS-CoV-related deaths were recorded as of March 5, 2016.^1^ The majority of reported cases originated from Saudi Arabia (2,121 cases). Also, MERS-CoV is believed to be of zoonotic origin and has been linked to camels in the Arabian area.^1,2^ In this report, the authors discuss the lessons learned from the MERS-CoV outbreak at King Abdul-Aziz Medical City-Riyadh (KAMC-R) from August through September 2015 from the Emergency Medical Services (EMS) perspective. The discussion includes the changes in policies and paramedic’s practice, the training and education in infection control procedures, and the process of transportation of these cases. The authors hope to share their experience in this unique situation and highlight the preparedness and response efforts that took place by the division of EMS during the outbreak.

## Event Identifiers

a. Event Type: Infectious Disease
b. Event Onset Date: September 2012
c. Location of Event: King Abdul-Aziz Medical City, Riyadh, Saudi Arabia
d. Geographic Coordinates: 24.7136° N, 46.6753° E
e. Dates of Observations Reported: August - September 2015
f. Response Type: Medical Relief

## Introduction

On December 2019, the first case of a novel coronavirus disease (COVID-19) was reported from Wuhan, China. After that, a world-wide outbreak has occurred. A global anxiety of medical personnel safety endures. A similar outbreak happened before in Saudi Arabia that started when Saudi Arabia had announced in September 2012 that a patient with acute pneumonia was found to have a novel beta-coronavirus (CoV);^1,2^ later, the patient had died from what is known today as Middle East Respiratory Syndrome Coronavirus (MERS-CoV).^3,4^ Since then, there have been clusters of local outbreaks that caused local emergency departments (EDs) to be closed, followed by a national emergency in 2014-2015. The MERS-CoV causes lower respiratory tract disease, such as pneumonia, which may be severe, especially in people with underlying comorbidities, cardiopulmonary disease, or weakened immune systems. Patients affected by this disease may present with the following symptoms: fever, cough, shortness of breath, gastrointestinal symptoms including diarrhea and nausea/vomiting, pneumonia, acute respiratory distress syndrome, and kidney failure.^5^

The recurrence of outbreaks and Saudi Ministry of Health (Riyadh, Saudi Arabia) policy of designating local centers to manage MERS-CoV patients have increased the demand on inter-facility transfers (IFTs). Patients with positive MERS-CoV in local hospitals are urgently transported by referring facility ambulance services to an identified “MERS-CoV center” and currently “COVID-19 center” to prevent outbreaks in local hospitals. The high mortality rate in MERS-CoV and high health-care-associated infections, including health care personnel acquired infections, required local hospitals to ensure that ambulance services personnel understand the risk of transporting such patients.

This article reported the overall experience of a Saudi hospital-based Emergency Medical Services (EMS) system with MERS-CoV: logistics of transfer, transfer policies, methods of monitoring personnel with CoV exposure, and training provided to EMS personnel.

## Settings

King Abdul-Aziz Medical City-Riyadh (KAMC-R), Saudi Arabia is a 1,501-bed tertiary-care hospital that includes a 150-bed ED that registers 250,000 visits per year; it is the largest trauma center in the region. The EMS department is a division of the department of emergency medicine; it is a hospital-based EMS service that provides 24 hour/seven days a week care, 365 days a year, under an on-duty board-certified emergency medicine consultant covering all KAMC-R facilities. The division has more than 100 personnel (Table 1). Minimum manpower shift requirements include one Advance Life Support (ALS) unit operated with at least one paramedic beside an ambulance driver/basic emergency medical technician, and two Basic Life Support (BLS) units operated with at least one basic emergency medical technician and an ambulance driver. In 2015, the services provided emergency and non-emergency coverage with total of 9,700 calls and with manpower of 106 employees working in the division, which has three subdivisions including operation, logistics, and administration. In 2019, the division transferred 17,402 patients. The ALS crew transferred 3,442 patients, of those 3,009 were emergency 911 patients. In addition, ALS crew transported 433 critical care IFTs. All the ALS units are equipped with portable ventilator and infusion pumps. Paramedics minimum skill competency is granted through the paramedic license requirements. All paramedics involved in IFT of critical patients have received advanced training in operating ventilators and syringe pumps; also, they are Advanced Cardiac Life Support, BLS, Prehospital Trauma Life Support, and Pediatric ALS providers.

**Table 1.** King Abdul-Aziz Medical City-Riyadh Emergency Medical Services resources

Total Employees	107
Medical Director	1
Operational Manager	1
Quality Officer	1
Educational Officer	2
Office Secretary	1
Emergency Medical Technician Basic (EMT-B)	55
Paramedic	33
Administrative Staff, Including Logistics	11
Ambulance Driver	2
All Vehicles (Including Logistic Vehicles)	38
Vehicles Type 3	14
Vehicles Type 2	21

## MERS-CoV Outbreak and Infection Disease Epidemic Plan (IDEP)

Early in 2013, KAMC-R launched the Infection Disease Epidemic Plan (IDEP), in response to the MERS-CoV outbreak, which consists of three phases that activate based on the number of confirmed cases reported to/within the hospital.^6^ However, on August 5, 2015, multiple cases were reported within the hospital, including patients and health care workers; Phase II of the IDEP was immediately activated alerting the outbreak within KAMC-R.^7^ Following that, the hospital medical director initiated major actions such as isolation rooms for suspected or confirmed case, implementing a screening process for all health care workers and new patients, education and training on identifying symptoms of the disease, and means of protection. Patients were categorized based on their presentation or confirmed diagnosis into three categories: low-risk patients, high-risk patients, and confirmed positive patients. The visual triage checklist for acute respiratory illness was utilized as a triage tool (Appendix 1; available online only). At that stage, EMS’s role began to increase for the aspects of transportation of some cases to different hospitals that are specialized for receiving MERS-CoV cases. Also, responding to emergencies that encounter symptoms of the MERS-CoV. By August 18, 2015, the number of confirmed cases increased, Phase III of the IDEP plan was activated, and the hospital completely shut down except for critical services; mutual aid agreements with other health care facilities were agreed upon to receive patients from KAMC-R. Moreover, a mobile field hospital was deployed outside of the ED to triage patients away to other health care facilities and to identify critically ill patients who required admission to the ED. Patients were transported via EMS to different destinations based on an algorithm used by dispatchers under medical direction (Appendix 2; available online only).

In addition to the non-MERS-CoV patients transported by EMS from the mobile field hospital and the ED, the EMS happened to transfer a total of 184 confirmed cases to the MERS-CoV center, the dedicated Ministry of Health quarantine, specialized in receiving and dealing with infectious diseases. Following the activation of Phase III of the IDEP plan, EMS focused on changing EMS high-risk practices, implementing immediate education and training, and changed the process of transportation for these specific cases.

## Changing the Practices of Prehospital Emergency Care

With the outbreak of MERS-CoV, there was great pressure on transporting patients to a previously identified MERS-CoV center where medical expertise and advance critical interventions such as extracorporeal membrane oxygenation (ECMO) were available. The EMS providers were anxious since little was known about this novel CoV. The EMS department moved quickly to implement certain practices with coordination with the infection control department. Mandatory steps implemented from the initial phase of being in contact with a suspected patient throughout the transportation process and upon return of the ambulance crew to the EMS station. Therefore, the first steps were prevention; this included early identification of any symptoms that might encounter patients or health care workers, screening the EMS providers at the beginning of each shift and after each transport through temperature checks, followed by tacking employees sick leave records. The second step was an assessment of the provider’s level of knowledge in this specific infectious disease through surveyed questions.

Along with that, in step three, certain medical protocols were updated and introduced, stopping selective procedures such as using nebulizer treatment on patient (metered-dose inhalers to be used if needed), drawing blood or intubation for patient, and using filter devices for ventilator equipment. Furthermore, all EMS providers were mandated to use personal protective equipment (PPE; ie, gowns, N95 masks, eye goggles, head covers, and disposable gloves) when attending to all cases. In addition, no food or drinking in the ambulances, even if there were no patients being transported. All PPEs and single-use materials were to be disposed of immediately in biohazard bags and bins after each transport. Following the recommendations from the Centers for Disease Control and Prevention (CDC; Atlanta, Georgia USA), the department generated a checklist to be used by all EMS providers prior to transporting a high-risk patient.^8^ Any EMS provider who had contact with a MERS-CoV case was monitored closely. They had to log in their temperatures every six hours and were instructed to report any flu-like symptoms. Ultimately, the emergency medical dispatcher (EMD) ensured complete information such as patient history, current status, safety of patient’s location, and the need for special resources. All EMS providers were cautioned that every patient could be a potential suspected MERS-CoV case (Appendix 3; available online only).

## Training and Education

With the MERS-CoV outbreak, EMS along with infection control acted immediately in the training and education aspects. Different training sessions were carried out using a variety of teaching methods such as frequent small-group classroom teaching, online videos, simulation, and demonstration sessions in how to use PPE, proper techniques of donning and doffing of PPE, and effective hand washing, hand hygiene, and hands-on practical sessions for mask fitting. Also, mandatory N95 mask-fitting sessions applied for all staff to ascertain the best-sized mask for each person along with training sessions on the use of Powered Air Purifying Respirator (PAPR) devices. Additionally, EMS acquired two patient isolation and transportation devices (ISO-PODs; TradeWays, LTD; Annapolis, Maryland USA) for complete patient isolation during transport. Numerous training sessions on this device were conducting including operating of the device, cleaning, and disinfection using high-fidelity simulation.

The education process and practical sessions were supervised by either the infection control officers or the EMS educators to ensure all personnel had the necessary skills and knowledge. Also, the operation leaders were monitoring the staff practice to ensure compliance rates among all staff. There were frequent updates about the MERS-CoV situation and visits from the infection control department to ensure proper progression throughout the outbreak.

Also, MERS-CoV was highlighted during the continuing education sessions for the EMS providers, covering the clinical presentations, epidemiological changes of the virus, new definitions or procedures, and risk factors were discussed. The EMS played an important role in educating the public about the virus with a focus on recognizing symptoms and proper techniques to prevent transmission of the virus; for example, proper handwashing and avoiding crowded areas. Also, they stressed the importance of making an honest declaration when a patient had contact with camels or had a recent history of travel.

## Transportation

With the declaration of the MERS-CoV outbreak in the KAMC-R and decision to shut down the hospital and transportation of patients to other hospitals, the EMS undertook this role and implemented new transportation schemes. First, EMS designated four ambulances (two ambulances for suspected MERS-CoV non-critical cases and two ambulances for confirmed MERS-CoV critical/non-critical cases) with one back up ambulance. These ambulances were striped out from supplies and hard equipment only to leave essential equipment. Based on the information and request provided to the EMD, the designated ambulance was deployed. For the confirmed cases, 6Mil plastic covers were placed in the ambulances covering the entire ambulance cabin for proper isolation. Also, for restriction of number of people in the ambulance, relatives were not permitted to accompany the patient in the ambulance, except for certain cases such as female or pediatric patient. In these cases, relatives were asked to sit in the front cab of the ambulance following the safety procedures. Any non-critical patient suspected or confirmed to have MERS-CoV were asked to wear a surgical mask.

During transport of any patient, the ambulance door between the driver cab and main cabin was always closed and the air condition was kept off, even though the ambulances have separate AC circuits. All ambulance windows were kept open to help with ventilation and circulating the air at the end of the call. In addition, at the end of every call, the providers were required to implement the cleaning and disinfecting procedure for their ambulance and equipment. The procedure included using the hydrogen peroxide aerosol fogger and leaving all equipment inside the ambulance and to ensure the locking of the ambulance for 60 minutes. After that, the provider would use antiseptic wipes for equipment and mop the floor of the ambulance with antiseptic solution. Then the vehicle would be aired, with all its windows and doors opened, for 30 minutes. All providers were mandated to follow the proper hand hygiene, hand washing, and use of PPEs with each steps of ambulance cleaning and disinfection.

## Monitoring Medical Status of Department Personnel

The outbreak of respiratory illness is terrifying. The EMS personnel needed an assurance that EMS division is supporting their well-being. After meeting with infection control and the medical director who was a board-certified emergency medicine physician, the following procedures were applied: daily body temperature (oral) monitoring to all EMS personnel at the beginning of each shift and hotline operated by infection control to address personnel inquiries. If a person was exposed, a clear pathway was implemented: the person was to report to employee health (during working hours) or to emergency flu clinic (out of working hours) immediately after the exposure, the person is granted an official sick leave, a nasopharyngeal swab would be acquired, a person was not allowed to return to work without the approval of the medical director, and pre-approval to access employees medical records was obtained (the only person allowed to access the record was the medical director).

## Discussion

The impact of a community outbreak of respiratory novel virus can be disastrous. The EMS agencies are regularly training their employees to function under extraordinary circumstances; however, once unique disasters occur, such as viral outbreak, additional effort is required to ensure that EMS will be functioning with maximum capacity during a disaster. The MERS-CoV is a serious life-threatening virus that has a high mortality (34.4%).^1^ It does not seem to be as infectious as COVID-19; however, it can be more lethal, the mode of transmission of MERS-CoV is human to human, and current data show that COVID-19 shares the same mode of transmission.^9^ Consequently, it is logical to conclude that measures taken during transporting MERS-CoV patients can be applicable to suspected COVID-19 patients. There were serious operational and administration issues with the first confirmed MERS-CoV patient. The hospital administration recognized that the longer the patient stays in the ED the more likely an outbreak will occur. An ISO-POD was utilized during the transfer of the first confirmed case. During the attempt to persuade the paramedic to take the patient, it was determined that the staff lacked the minimum infection control techniques needed for such transfer. It turned out that they were not familiar with the ISO-POD and it was their first time to operate such device. The extensive training course to all EMS personnel was conducted in a week’s time, and after that, paramedics dealt with a total of 184 confirmed positive MERS-CoV patients without significant issues. Paramedics are health workers, and with proper training, they can manage such transfers. The EMS continuing education and disaster drills might not cover the whole spectrum of emerging novel respiratory illness outbreaks. Unique disasters may prevent establishing an internationally recognized recommendation on prehospital continuing education. In this experience, it was clear that paramedics needed training, and once provided, they were confident and competent in transporting critically ill positive MERS-CoV patients. After reviewing the literature, it was recognized that this program must be designed based on the special needs and with clear justification.^10^ The result of that program is EMS personnel that are able to manage MERS-CoV. Later, during the COVID-19 outbreak, they were confident and prepared to transport patients. It is reasonable to discuss training needs with EMS staff before implementing such programs.

Occupational safety is a multidisciplinary effort that includes employees and communities.^11^ The division recognized that an outbreak of MERS-CoV to the team members would have disastrous consequences, including the possibility of closing the EMS division and failure to respond to any emergency calls. The concern was clearly delivered and discussed with all stakeholders, including hospital administrators and paramedics. The decision was to ensure that enforcement of policies was implemented; also, the compliance was monitored on a daily basis. In addition, paramedics were informed that all measures were taken to protect them, and their compliance was the key to success or fail on this matter. The employees were assured that medical care will be provided immediately in case of exposure. Every MERS-CoV positive patient was pre-planned by transporting team, medical director, sending unit, and receiving unit. The high-risk calls were audited on a daily basis by division quality officer and disciplinary actions were taken. Paramedics were required to record their body temperature at the beginning of every shift and strict compliance was monitored by division operation chief. The pre-determined goal in response to MERS-CoV outbreak was to have zero infection acquired to paramedics. There were three paramedics removed from service due to possible exposures and a nasopharyngeal swab was taken before they were allowed back to service. It cannot be concluded that the rate of zero acquired infection was related to only these policies; however, the assurance of all stakeholders, including providers, that measures were taken and audited was an assistance to achieve success. It is the recommendation that EMS agencies train, enforce, and monitor their compliance to achieve the pre-determined goals.

## Limitations

This report has some limitations. The paper describes only one service (EMS) from the whole experience against MERS-CoV. In addition, this report and experience was limited to one population. Further, this report lacks the patients’ outcomes.

## Conclusion

The outbreak was a true test to the system; it outlined the weakness in EMS, as well as the need for proper preparedness and planning. The utilization of the available evidence in the field of EMS and previous experiences had an impact on the outcome of the outbreak. This epidemic experience showed the importance of education, communication, employee support, and planning. On October 18, 2015, the outbreak was contained and the IDEP was officially deactivated; due to the dedication of the staff, there was no EMS personnel affected by MERS-CoV during the outbreak.

## Supplementary material

For supplementary material accompanying this paper visit http://dx.doi.org/10.1017/S1049023X20000709. click here to view supplementary material
