# Supplementary material for: The Middle East Respiratory Syndrome Coronavirus (MERS-CoV) Outbreak at King Abdul-Aziz Medical City-Riyadh from Emergency Medical Services Perspective
Source: Prehosp Disaster Med. 2020 May 20:1–5. doi: 10.1017/S1049023X20000709 (PMC7264457; doi:10.1017/S1049023X20000709)
Supplement: Supplementary file 1 [file S1049023X20000709sup.zip › S1049023X20000709sup001.pdf]

APPENDIX B

Visual Triage Checklist

Visual Triage Checklist for Acute Respiratory Illness

Date: Time MRN:

Name: ID#: Hospital:

	Points (adults)	Pints (children)	Score
A. Clinical symptom/sign			
Fever	2	1	
Cough (New or worsening)	2	1	
Shortness of breath (New or worsening)	2	1	
Nausea, vomiting, diarrhea	1	-	
Sore throat and/or runny nose	1	-	
Chronic renal failure, CAD/heart failure	1	-	
B. Risk of exposure to MERS			
Exposure to a confirmed MERS case in the last two weeks	3	3	
Exposure to camel or products (Direct or indirect*) in the last two weeks	2	2	
Visit to a healthcare facility that had MERS case in the last two weeks	1	1	
**Total Score**			

* Patient or household

A SCORE ≥ 4, PLACE PATIENT IN AN ISOLATION ROOM AND INFORM MD FOR ASSESSMENT

MERS COV TESTING SHOULD BE DONE ONLY ACCORDING TO CASE DEFINITION

Staff name: ______________________ ID number: ___________________
