# Supplementary figures and images for: The Middle East Respiratory Syndrome Coronavirus (MERS-CoV) Outbreak at King Abdul-Aziz Medical City-Riyadh from Emergency Medical Services Perspective
Source: Prehosp Disaster Med. 2020 May 20:1–5. doi: 10.1017/S1049023X20000709 (PMC7264457; doi:10.1017/S1049023X20000709)

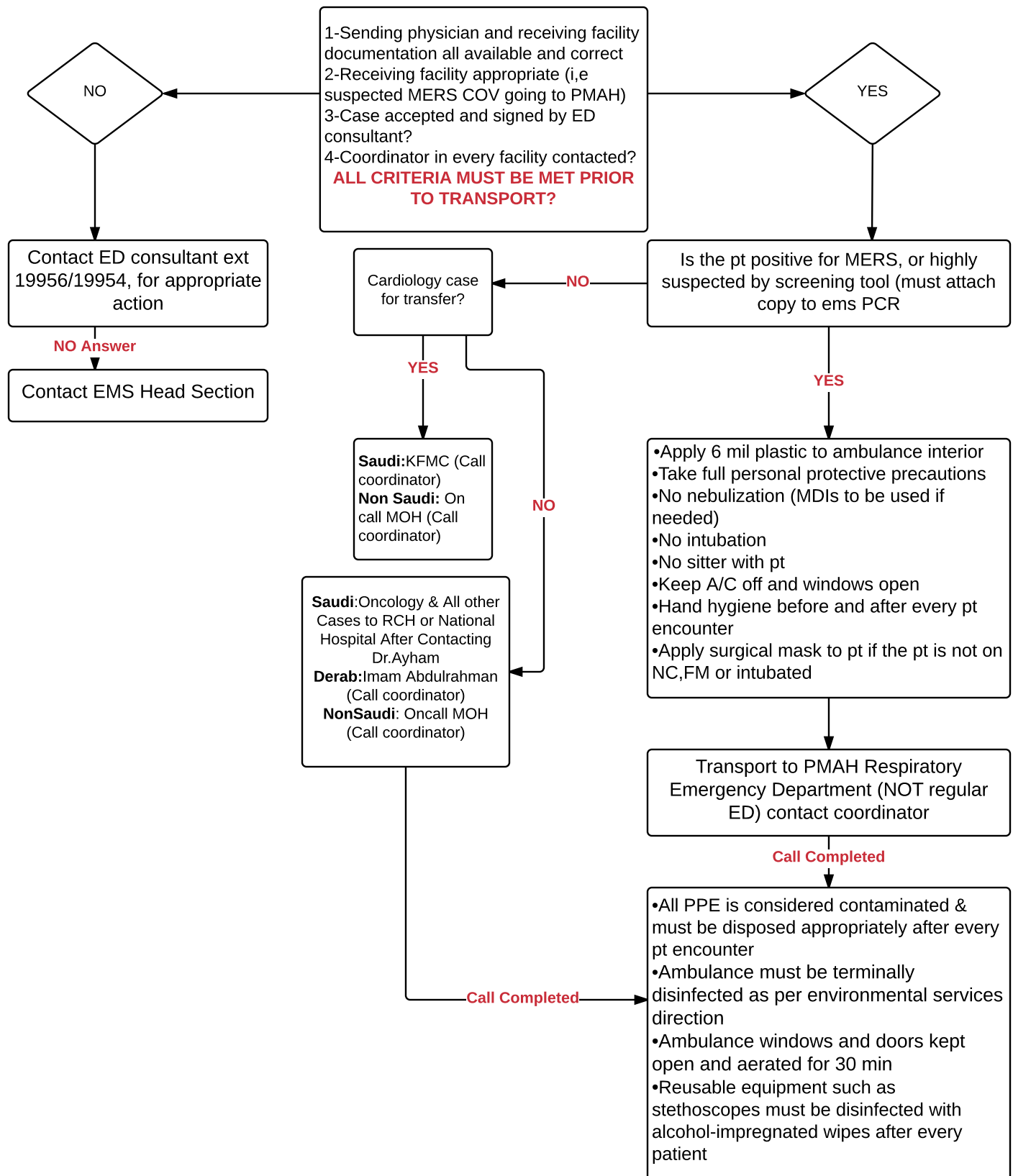

Supplement: Supplementary file 1 [file S1049023X20000709sup.zip › S1049023X20000709sup002.pdf]

**Dispatch questions/History taken By EMS**

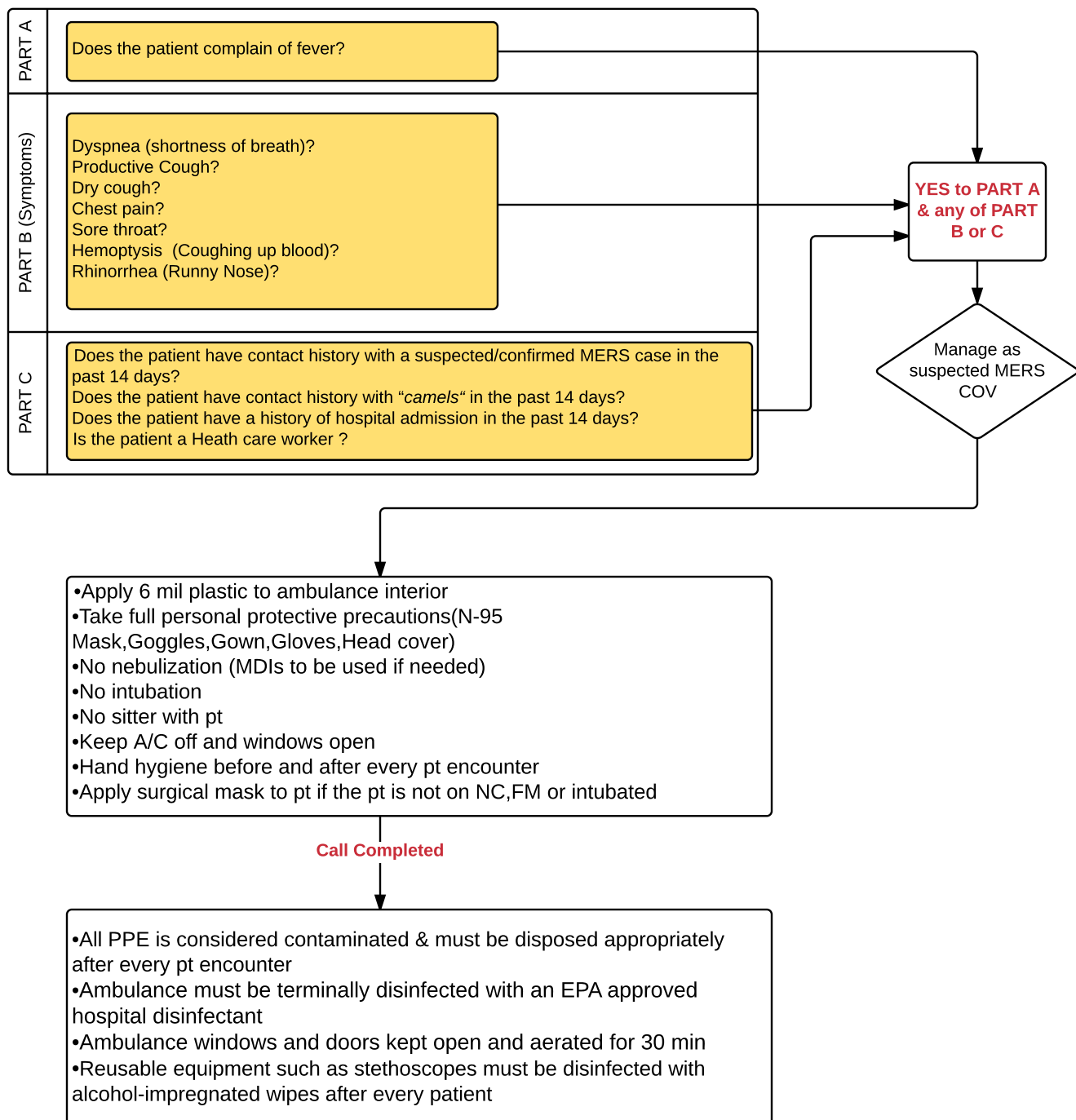

Supplement: Supplementary file 1 [file S1049023X20000709sup.zip › S1049023X20000709sup003.pdf]
